# The nature of the open birth interval distribution

**DOI:** 10.12688/gatesopenres.13177.1

**Published:** 2020-10-07

**Authors:** John Ross, Kristin Bietsch

**Affiliations:** 1 Independent Demographic Researcher, New Paltz, New York, USA; 2 Avenir Health, Glastonbury, Connecticut, 06033, USA

**Keywords:** Fertility, Open Birth Intervals, Parity, Models, Family planning, Developing countries

## Abstract

**Background**: The open birth interval -- the time since the woman’s latest birth -- is closely correlated to the usual fertility measures, but it adds important information from the age of the woman’s youngest child, with its implications for her freedom from domestic roles.  Studies of the open interval by age and parity can elucidate the transitions in reproductive behavior that women experience over time.

**Methods**:  249 surveys of married women in 75 countries in the DHS series provide information on the open  interval by age and parity, and by the  fertility measures of the total fertility rate (TFR), the general fertility rate (GFR), and children ever born (CEB), with time trends.  Stata 15 and the “R” software were used, and a two-parameter equation was employed to model the distribution.

**Results**:  The distribution of women by the open interval follows a downward curve from birth to 20 years; it varies across countries and over time only by its starting level and the steepness of the curve. Declines in the shortest intervals soon after birth reflect recent fertility declines. Variations are large by both age and parity, but in quite different patterns. Past modeling analyses demonstrate the effects of female and spouse mortality, declining fecundability, contraceptive use, and reduced sexual exposure. Both period and cohort effects can impact the curve. The open interval distribution is modelled in an equation with two parameters and calculated for the latest surveys in the 75 countries.

**Conclusions:** The time since a woman’s birth is easily captured with a single question in successive surveys. Changes in the open interval distribution serve as sensitive indicators of recent fertility changes, and the dynamics of reproductive behavior across women’s life stages are captured in new ways, as gauged by age and parity trends in the distributions.

## Introduction

The open birth interval – the time since a woman’s most recent birth – has been of interest since the early 1960s for its relation to closed intervals, for its relationship to fertility rates, and for its use as a way to trace changes in contraceptive use and the effects of family planning programs. In addition, the changing roles of women for activities outside the home, as the open interval grows and the youngest child ages, have been studied. Modeling exercises have investigated some of these features over the years, including the effects of age structure upon the open intervals. However, the modeling work has been seriously hampered by the lack of empirical data on large sets of data. Here we explore the nature of the open birth interval distribution based upon a set of 249 surveys in 75 countries.

We proceed with two major parts of the article, first to look back at past analyses of the open birth interval, with its extensive modeling work. Much of that work has been forgotten and deserves a fresh review, and it helps shape the focus of the new work here. Second, against that background, we examine information on the empirical patterns that emerge from a large set of national surveys containing extensive data not available in the past, with particular attention to the interactions of the open birth interval with age and parity.

Efforts to model the open birth interval go back more than 50 years to the pioneering work of Srinivasan, 1968 and Srinivasan, 1970 and Sheps *et al.* 1967. Sheps was one of the first to apply life table methods to the movement of women from one birth toward another, recognizing that some women would never go on to a next birth. Srinivasan, in a series of articles starting in 1966, explored relationships between closed and open intervals in their connections to fertility and their potential for detecting family planning program effects. His initial work (1966) suggested that changes in the open interval could serve as an indicator of recent fertility change, but Sheps *et al.* (1970) countered by noting the dangers of truncated observation times in survey data, especially when the analysis was parity specific. Subsequently Srinivasan (1968) compared alternative models of the open birth interval and its relation to fertility with allowance for female or spouse mortality, by parity. He assumed the parity progression ratios and the parity-specific birth rates to be constant over time, except that under a further scenario, those rates followed a linear function of change. In this work he examined three different groups: women reaching the next parity, women failing to do so who live through the full reproductive period, and women failing to do so who suffer death of self or spouse. The weighted mix of the three types yielded the overall results.

Srinivasan (1970) also conducted correlational analyses to relate mean interval lengths, both closed and open, to parity, separately by age group. Using data from a 1965 survey conducted in the Gandhigram, India, area he finds that mean interval lengths, whether closed or open, are subject to counter influences between age and parity: later ages tend to lengthen intervals, but higher parities tend to shorten them. Age mattered systematically in that below age 30 open intervals were shorter than closed ones, but above age 30 open intervals were longer, regardless of parity. Reproductive behavior shifted toward the avoidance of births as women aged. Parity itself correlated closely, and negatively, with the open interval, much closer than with the closed interval, rising across the age groups to above 0.50 after age 30.

Hastings & Robinson (1975) replicated Srinivasan’s work on Gandhigram data to compare it to white U.S. women in a 1970 U.S. national sample. They confirmed that mean interval lengths, within each parity group, rose regularly by age for closed intervals, and did so quite dramatically for open intervals. Correlations were even closer with marital duration, a better indicator of reproductive exposure, than with age at each parity. Again, these correlations were higher with open than with closed intervals. In a three-way breakdown of respondents, the interval lengths rose by age within each marital duration group and vice versa, but they declined by parity within each age/marital duration group, reflecting the probable necessity to have short intervals in order to reach a high parity in a limited time. Finally, interval lengths were related to the (rather insensitive) fertility indicator of children ever born (CEB) at each age, showing agreement with Srinivasan’s finding of negative associations between high fertility and short open intervals.

Assuming a marital duration of ten years, Pandey & Singh (1988) offered a simple model that generated open birth interval lengths. Parity differences were deliberately ignored to offer an approach that would allow for the frequent problem of data sets too small to compare multiple determinants. They employed data from the 1969–70 rural survey in Varanasi, India, and obtained a good fit of open interval lengths between the survey and the model.

The effects of variations in fecundability and sterility according to parity were explored by Venkatacharya (1972), Venkatacharya (1988); his model projected a single marriage cohort forward, examining effects on the open interval without relating it to fertility change. The mean open interval lengthened with marital duration but diminished as parity increased. As a cohort moved forward, the open interval was modified by diminished fecundability, more sterility, and more contraceptive use with better contraceptive effectiveness. He noted that comparisons across countries or through time were feasible by standardizing the parity distribution. He noted Cox’s conclusion (1962) for renewal theory that, under restrictive assumptions, either the open or the closed interval can be derived from the other; however, he did not pursue the connection.

Later, Tiwari & Dwivedi (1994) addressed the question of variability in the open interval distribution due to declining fecundability and increasing secondary sterility after a last birth, examined for separate age groups and parity progression ratios. Comparisons of model results from the Varanasi survey of 1969–70 and from two synthetic models showed close agreement, with little sensitivity of the open interval distribution to fecundability changes. Their use of renewal theory let them estimate the open interval backward from the closed interval.

Yadava *et al.* (2013) used closed and open birth intervals to estimate parity progression ratios, modifying earlier work (especially by Yadava & Bhattacharya, 1985) to allow for cases of rapid fertility declines, as in some states of India. Data from three rounds of the National Family Health Survey (NFHS) were employed (1992–93, 1998–99, and 2005–06) to test new procedures that yielded better estimates of the parity progression ratios compared to earlier work.

Schmertmann (1999) noted the use of both last births and prior births to enlarge the observation base for greater reliability of the fertility estimate, providing that age-specific fertility rates have been unchanging. He illustrated this over five years for Sao Paulo from the 1991 Brazilian census and found that sensitivity tests showed only minor biases under violations of the assumptions of constant fertility and homogeneity of fertility rates within age groups.

Then Schmertmann & Caetano (1999) extended that work. They used open interval information to produce fertility estimates in the Coale & Trussell (1974) model of age-specific marital schedules, again with Brazilian census data. Using the date of the last birth within the previous five years enlarged the observation base, improving the reliability of small sample estimates. They obtained Coale-Trussell *M* and *m* estimates for 723 municipalities in Minas Gerais State and demonstrated the superiority of using date of last birth over births in the last year for producing the key parameter estimates in the Coale-Trussell model. The 1991 Minas Gerais data were chosen as a test case because of earlier work by Assunção *et al.* (1998), who had used Bayesian spatial smoothing of data on births in the previous year to assess municipal-level fertility control. (Schmertmann notes that many censuses have included a question on the date of the last birth, at least as of 1999, in his review of that year.)

The open interval distribution was shown to parallel the age distribution in a general population by Feeney & Ross (1984). Using 1976 national Indonesian data they showed how both distributions provide snapshots of women surviving from an entry point of births, one being watched for delay to another birth and the other for delay to death. In populations that are stable or stationary the open interval distribution can be used to estimate closed intervals, permitting estimates of fertility. Absent a stable population, however, fertility estimates are dubious. Estimating fertility from an open birth interval distribution is analogous to estimating mortality from an age distribution, and this is impractical unless the age distribution has a fairly simple structure. The analogy between a population age distribution and the open interval distribution was noted by Sheps & Menken [1973]; it was also suggested by Srinivasan [1970]).

A general review of much of the literature to 2015 is given by Singh (2016); he also presents a model with both open intervals and closed intervals, including those that “straddle” (starting before and extending beyond) the survey date, and “forward” intervals (starting with the survey date and extending to the next birth). He used data from the Varanasi, India, survey to examine differential probabilities of conception near the end of postpartum amenorrhea, with constant fecundability thereafter, and with a random variable controlling the interval between the termination of postpartum amenorrhea and exposure to the risk of conception.

The modeling literature gives little attention to contraceptive use or to policy uses of open birth interval information, including any effects of family planning programs. However, in probably the most sophisticated study so far, Towriss & Timaeus (2018) used repeated surveys in four east African countries (Ethiopia, Kenya, Tanzania, and Zimbabwe) to measure changes in open and closed intervals and their relationships to the use of contraception and to fertility rates specific to birth interval lengths. Their methods control for parity and age, changing interval lengths over calendar time, and whether subjects ever married.

There has been nearly no experimental work to relate program effects on interval lengths. In one exception, open intervals shorter than 30 months proved to be the best predictor of contraceptive adoption over a two period in a Korean study, better than a variety of other personal and demographic determinants ( Ross & Bang, 1966). At about the same time, a non-experimental survey of women aged 30–39 in Taiwan ( Mohapatra, 1966) showed contraceptive practice helping to extend the open intervals.

In review, some models have been concerned with finding a few parameters to capture the distribution of the open interval, some of them parity specific. Others have focused on the open interval as an index of fertility levels and change. Parity progression ratios have been part of a few models, as well as allowance for distributions by age and marital duration or both. Some have allowed for the effects of postpartum infecundability or heterogeneity of conception risk. Rather few publications have tested the models against actual data. One model has been fitted to the open birth interval distributions ( Ross & Bietsch, 2019); it is compared here to three alternatives. The focus is directed especially to the interactions of interval lengths with age and parity, and to parity changes within age groups. Four case studies examine historical influences and trends, and the interval lengths are correlated to fertility.

Specifically, modeling has been used:

- to explore the relation of open intervals to closed ones, and ways to estimate each from the other
- to explore the utility of open intervals as indices of fertility and fertility change, by using technical modeling work, with attention to specific age, marital duration, or parity subgroups
- to explore how they can reflect a diminution in the risk of conception through declines in fecundability, mortality of spouse, or decreased sexual exposure
- to investigate how they might measure changes in contraceptive use and the effects of family planning programs

## Methods

We draw on national surveys in the Demographic and Health Survey (DHS) series ^1^ from 75 countries, with repeat surveys in many, permitting time trend assessments. The DHS series is implanted through collaboration with a large number of countries, primarily those in the developing world. It is now comprised of over 250 household surveys taken from the late 1960s to the present, in over 80 countries, with sample sizes varying from about 5,000 to 30,000. Participants are selected by scientific sampling methods from sample frames in each country, in cooperation with local experts, for example in census bureaus. Surveys are typically conducted about every five years to allow comparisons over time. Data are presented both nationally and by sub-national reporting area. The DHS series is available free, with open access and is available online. Full documentation is available at https://dhsprogram.com/What-We-Do/Survey-Types/DHS-Methodology.cfm.

All available DHS surveys were included in our analysis, excluding only those lacking information on the time since the woman’s latest birth. As a way to include more countries with the required data, only married women were studied. The 75 countries in the analysis and survey dates appear in Table 4. The variables included in our analysis are the date of each survey, the time since the woman’s latest birth, her age and parity when interviewed, and whether she was pregnant.

The open interval distributions were generated both overall and specific to women’s age and parity. Currently pregnant women were included in the first-year interval, being close to birth and having very similar characteristics to the first-year group.

Special processing of the survey records using the “R” and Stata 15 software yielded the distributions for each survey. The needed variables were extracted, and the distributions tabulated from birth through the 20th year. The software we used was the RStudio Version 1.2.5019 of the “R” software ( RStudio Team, 2020) and the State/SE 16.0 for Windows (64-bit x86-64) for Stata ( StataCorp, 2019). Details of code for reproducing the analysis can be found in the *Data availability* section ( Bietsch, 2020).

For most results, simple summaries with averages and distributions are shown. The *Modeling the open birth interval curve* exercise uses an exponential equation with two parameters.

## Results

We present first the common shape of the open birth interval distribution as found in every survey, and then present variations across time, countries, age, and parity. Four country cases are also included.

**Figure 1.**
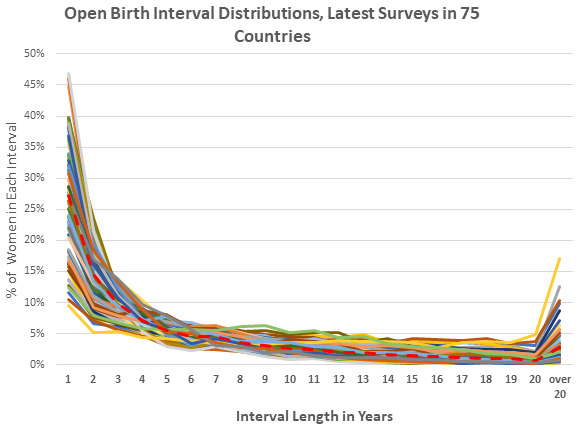
Open birth interval distributions, latest surveys in 75 countries.

The average pattern for the distribution ( Figure 1, dotted line) starts high for women either pregnant or in the first year after birth. It descends sharply and regularly toward the twentieth year, i.e., for women who have gone twenty years since their last birth. This descent partly reflects the close relation (below) between women’s age and the interval length, as well as the fall-off in numbers of older women, as in the population pyramid.

Variations across countries in Figure 1 show that the shape of the curve, with the regular fall-off toward longer intervals, is common. However, countries vary greatly in their placements within each interval and in the steepness of the curve. In the first interval the percentages range widely; the lowest point is for Ukraine at 10% and the highest point is for Niger at 47%. (Because the distribution totals 100% for each country, a curve starting above the average is followed by points below the average.)

**Figure 2.**
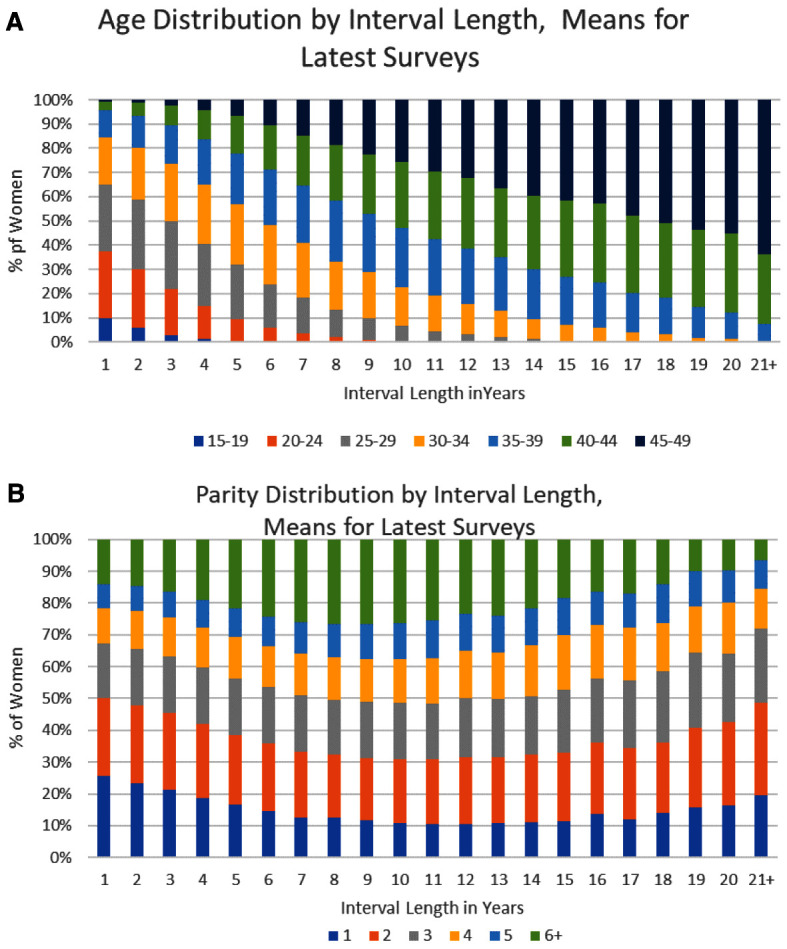
**A**) Age distribution by open interval. **B**) Parity distribution by open interval.

Variations according to age and parity are examined in Figure 2. Both characteristics figured in many of the models reviewed, often to refine the relationships between the open interval and fertility indices. Few models were tested against open interval data, available then in only a few places. The figure displays the actual age and parity patterns, which are quite dissimilar.

Women’s ages vary systematically across the intervals. The four age groups 15–34 gradually vanish, while the three oldest groups grow until women over age 40 account for over 93% of the total ( Figure 2, last two bars). The average age rises in step with the interval length; the correlation between mean age and interval length is R2 = 0.92.

However, the parity pattern differs. It too is systematic, but the mix of parities varies in unexpected ways as intervals lengthen. Most notable is the U-shaped curve for women at parities 6 and above. In the middle intervals they account for about a fourth (up to 27%) of women, but by the end they fall to a mere 7%. The levels for parities 4 and 5 stay within smaller ranges: parity 4 starts at 11%, rises to a steady 14% to 17%, and ends at 13%. Parity 5 begins at 8%, levels off at 11% to 12%, and ends at 9%.

Meanwhile parities 1 and 2 together hold the largest shares in the first five intervals. Then parity 1 declines while parity 2 stays level at about 20%. Both gain somewhat in the final intervals, as parity 3 also does, though until then parity 3 is nearly constant at 18%.

These changes reflect conflicting tendencies. A timing limit helps explain the middle hump for parities 6+. Having six or more children uses up much of the childbearing time, so few women will still have a large gap left over since the latest birth. That constrains the proportion who are found in the final intervals. A counter tendency is that high fertility women tend to have short birth intervals and a recent birth, which would drive up their share in the early intervals, but there they face competition from the early childbearing of younger women at short marital durations. Such cross currents produce the patterns shown in Figure 2.

A remarkable finding in Figure 2 concerns the emergence of smaller families, well below traditional fertility levels. From interval 7 through interval 14 the sum for parities one through three is 49% or 50%, half of all married women. The percentage then rises steadily to a full 72% in the final interval. After approximately interval 7 few women will have another birth, since most births that ever occur do so within about one to seven years of a previous birth. The persistence of the 50% and higher level reflects past growth in contraceptive use, as well as the emergence of secondary sterility with aging and reduced sexual exposure. The longer the interval, the longer has past behavior avoided an additional birth.

Parity by age: how does the parity pattern change as women age, and how does the pattern change when fertility declines? Parts of the modeling literature focused on this, especially as noted above by Srinivasan (1970) and Hastings & Robinson (1975). As women postpone the next birth or avoid it entirely, their open intervals lengthen, fewer move on to the next parity, and fertility rates decline. This is illustrated over 25 years for Colombia ( Table 1), where the total fertility rate (TFR) between 1990 and 2015 fell from 2.8 to 2.0 and contraceptive prevalence rose from 66% to 81%.

**Table 1.** Percentage distribution of women by parity within each age group, Colombia, 1990 to 2015.

Age groups	Parity								
15–19									
	**0**	**1**	**2**	**3**	**4**	**5**	**6+**	**Total**	**Mean**
1990	41.6	48.7	7.1	0.9	1.6	0.0	0.0	100	0.72
1995	33.7	51.5	12.5	1.8	0.0	0.4	0.0	100	0.84
2000	32.3	53.6	12.9	0.6	0.6	0.0	0.0	100	0.84
2005	34.8	47.9	15.9	1.3	0.1	0.0	0.0	100	0.84
2010	36.1	52.0	10.7	1.2	0.0	0.0	0.0	100	0.77
2015	39.3	49.1	10.5	1.1	0.0	0.0	0.0	100	0.73
20–24									
	**0.0**	**1.0**	**2.0**	**3.0**	**4.0**	**5.0**	**6+**	**Total**	**Mean**
1990	14.6	44.1	29.0	7.5	3.1	1.4	0.2	100	1.46
1995	16.8	40.5	26.9	11.4	3.5	0.7	0.2	100	1.47
2000	13.0	44.3	28.7	10.9	2.1	0.8	0.1	100	1.48
2005	16.6	44.9	26.5	8.4	3.1	0.5	0.1	100	1.38
2010	17.9	47.5	24.6	7.8	1.8	0.5	0.0	100	1.30
2015	19.6	48.8	22.4	7.7	1.3	0.2	0.0	100	1.23
25–29									
	**0.0**	**1.0**	**2.0**	**3.0**	**4.0**	**5.0**	**6+**	**Total**	**Mean**
1990	8.7	24.7	30.6	18.0	13.0	3.6	1.4	100	2.19
1995	8.7	28.8	30.3	19.1	8.7	2.6	1.8	100	2.05
2000	7.8	26.7	34.9	19.2	7.8	2.3	1.3	100	2.05
2005	9.1	30.7	31.4	17.4	7.2	2.8	1.4	100	1.97
2010	11.5	32.2	33.8	14.3	5.8	1.7	0.7	100	1.79
2015	12.8	34.6	33.3	13.6	3.8	1.4	0.6	100	1.67
30–34									
	**0.0**	**1.0**	**2.0**	**3.0**	**4.0**	**5.0**	**6+**	**Total**	**Mean**
1990	2.2	13.3	28.7	22.6	15.8	10.6	6.9	100	2.97
1995	5.1	15.5	31.6	22.0	13.3	6.0	6.5	100	2.68
2000	5.1	17.1	34.4	22.6	12.0	5.3	3.5	100	2.50
2005	4.5	17.6	33.5	24.4	11.2	4.8	4.0	100	2.51
2010	5.5	22.0	37.0	19.8	9.2	4.1	2.4	100	2.27
2015	7.1	23.5	39.5	18.8	6.6	3.0	1.6	100	2.10
35–39									
	**0.0**	**1.0**	**2.0**	**3.0**	**4.0**	**5.0**	**6+**	**Total**	**Mean**
1990	4.4	10.3	22.6	25.1	14.0	7.7	16.1	100	3.25
1995	2.4	7.8	24.5	25.1	15.8	11.5	12.9	100	3.33
2000	3.6	11.5	27.8	27.9	14.4	8.0	6.8	100	2.91
2005	2.4	11.2	32.3	27.1	12.5	6.2	8.2	100	2.89
2010	3.1	13.9	34.6	25.2	12.0	5.7	5.5	100	2.69
2015	3.7	17.8	38.1	23.2	9.5	4.0	3.7	100	2.45
40–44									
	**0.0**	**1.0**	**2.0**	**3.0**	**4.0**	**5.0**	**6+**	**Total**	**Mean**
1990	2.5	5.2	16.6	17.9	16.3	11.1	30.5	100	4.02
1995	2.2	4.6	18.5	25.7	17.8	10.4	20.8	100	3.71
2000	2.7	6.2	24.6	24.0	17.3	11.3	14.0	100	3.40
2005	2.3	8.4	27.0	27.9	15.7	8.5	10.2	100	3.15
2010	2.9	9.4	30.0	28.3	14.0	6.8	8.6	100	2.97
2015	3.7	11.6	34.4	25.7	10.9	6.7	7.0	100	2.78
45–49									
	**0.0**	**1.0**	**2.0**	**3.0**	**4.0**	**5.0**	**6+**	**Total**	**Mean**
1990	2.8	5.0	7.3	10.9	10.7	13.3	50.0	100	4.72
1995	2.6	4.7	13.3	20.6	19.1	10.3	29.4	100	4.03
2000	2.6	5.0	17.4	23.3	16.1	12.7	22.9	100	3.80
2005	3.1	6.7	21.2	27.3	17.7	9.6	14.4	100	3.39
2010	2.7	7.6	27.6	27.3	16.2	8.1	10.6	100	3.15
2015	3.3	10.3	29.7	28.9	13.6	6.1	8.2	100	2.92

Over time, the average parity of women declined, but less so at ages 15–19 where about a third were still at parity zero with another half at parity 1 ( Table 1 first two columns). The declines were very regular in the other age groups, and they grew sharply as age increased. At ages 20–24 mean parity fell from 1.46 to 1.23, or a 0.23 decline; in subsequent age groups the declines grew to 0.52, 0.87, 0.80, 1.24, and finally to 1.80 at ages 45–49. In short, high parity births were disappearing during the same decades as contraceptive use was growing.

Parity *distributions* shifted in interesting ways that lie behind the changes in the means. The early concentration of the oldest women at the highest parities of 6 and above nearly vanished over the next 25 years, dropping from 50% of women to only 8%, and for women 40–44 from 30% to 7%. Declines in parities 4 and 5 are evident by ages 25–29.

Meanwhile, among younger women the lower parities were gaining, but differentially. At ages 15–19 parities 0 and 1 accounted for most women with shares that changed little over time. But parity 0 showed an upward trend in the recent surveys starting at ages 20–24 and continuing in each age group through 30–34, as did parity 1 at all ages through 45–49. Parity 2 began to grow at least at ages 30–34 and above. Parity 3 followed a rather irregular trend pattern, losing shares in the recent years at ages 25–29 and 30–34.

Primary sterility of either spouse is reflected at the 3% level from ages 35–39 onward.

Colombia serves here as an example during a rapid decline in fertility. The data on other countries with large fertility declines and large extensions of the open interval give a similar picture, while countries with small changes in fertility or in their open intervals show much more modest modifications of the parity patterns.

### Country cases

We examine the four country cases below to illustrate different patterns and historical influences. Compared to these cases, China can serve as the most extreme example of a policy impact on the open interval distribution. We lack the open interval data but the major events are well known ^2^. With China’s imposition of its one-child policy in 1979, births at parities one and above began to disappear, although there were exceptions to the policy, e.g. for minority groups and for some families with only a daughter. Most women at higher parities moved into ever longer open intervals. The absolute numbers of births also declined, producing a change in the size of the first open interval that over time tended to flatten the curve. The sudden and strong period effect of the policy set off cohort changes, in which each new set of married couples would trace a different path of childbearing from that of their parents.

**Figure 3.**
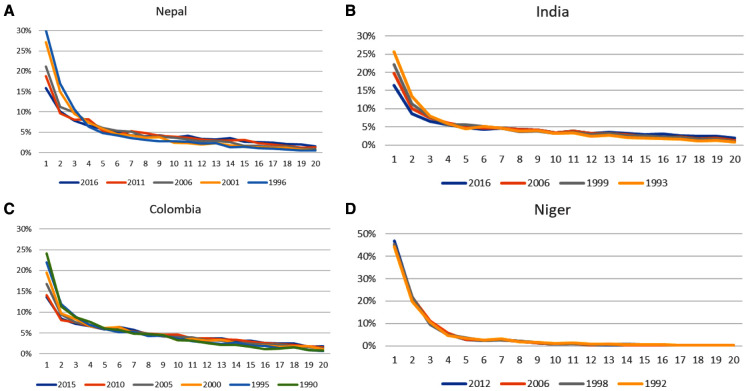
Changes over time in the open interval distribution. **A**) Nepal, **B**) India, **C**) Colombia, **D**) Niger.

Four rather different country experiences are shown in Figure 3; these are chosen somewhat arbitrarily for diversity in location and in degree of change over time: for Niger in sub-Saharan Africa, Nepal and India in Asia, and to give more information for Colombia in Latin America.

Niger is a case quite contrary to China, one with no official restrictions on childbearing, high desired family sizes, and high fertility rates. Consequently, women are clustered toward short open intervals. Both period and cohort effects have been small, with little change in the open interval curve across the surveys taken between 1992 and 2012. Nearly half of women are either pregnant or within a year of their latest birth. At the other extreme, Nepal has had the fastest decline in that respect, experiencing a remarkable rise in contraceptive use over the 20 years from 1996 to 2016, nearly doubling its use, from 28% to 53%. India presents the case of a very large and diverse country that overall has shown a regular decline in the size of the first interval, from 26% to 17%, over 25 years. If data by state were available the extent of diversity across such states as Bihar, Gujarat, and Kerala would be of considerable interest. Finally, Colombia has experienced the greatest transition in childbearing. Over the 25 years from 1990 to 2015 the percentage pregnant or in the first year after birth fell from 24% to about 14% where it has stayed over the last two surveys taken five years apart. Period effects have included the increased availability of contraceptive methods through a variety of channels and methods. The discussion above for Colombia provides related trends by age.

All four countries show the typical descending curve by interval, but at different starting levels and slopes. The most sensitive part of the curve is at the start, and its pace of change downward has varied. The declines per year for the percentage in the first interval ( Table 2) show Niger and Nepal at the two extremes and Colombia and India in between.

**Table 2.** Annual change in percentage of women in first year after birth, select countries.

Country	Annual change in percentage of women in first interval
Colombia	-0.42
India	-0.41
Nepal	-0.71
Niger	-0.12

### Influences upon the level and shape of the open interval distribution

In review we can list major influences that affect the form of the open interval distribution. In addition to these there are ad hoc effects from idiosyncratic forces of a temporary nature.

- The age distribution of women in the population is a key influence. As reflected in the population pyramid the numbers decline substantially with age, and they do so also in the later open intervals due to the close correlation between age and intervals. Therefore, irregularities in the population age distribution will disturb the open interval distribution. Because the number of women diminishes rapidly with the interval length, the trajectory for every age group and parity group declines.
- The fertility rate acts as a major determinant of the tilt of the open interval distribution; the higher the fertility rate, the higher the first-year percentage of women.
- Female mortality depresses the number of women involved, and it does so differentially by interval, again reflecting the advancing age of the women ^3^. Note that spousal mortality leaves the women in the population but tends to reduce their birth rates, prolonging their birth-free time.
- The onset of reduced fecundability of either spouse becomes more important with age.
- Contraceptive use, both its level and its varying effects according to the method mix, prolong both closed and open intervals. A related influence is the lack of sexual exposure due to spousal absences. The longer open intervals can especially reflect the joint forces of increased contraceptive use, decreased fecundability, and decreased sexual exposure

In general, the shape of the distribution is a function of changes occurring in the past that have impacted childbearing, with both period and cohort effects. Period effects reduce births during disasters and depression but increase them for example when men return from a war, with many births following new marriages. Those effects shift the distribution of women toward longer intervals in the first case and toward shorter ones in the second case. Cohort effects can be quite marked when fertility falls rapidly. Each new cohort of births is smaller than the previous one, changing the start of the distribution. Each cohort feeds reduced births into the next interval in a systematic pattern as the fertility rate continues to fall. That transition produces a series of overlapping curves, or waves, moving forward until a new stable state emerges after fertility finally plateaus. Under other scenarios irregular waves can appear in the curve if fertility has varied irregularly.

### Relation of the open birth interval to fertility rates

**Figure 4.**
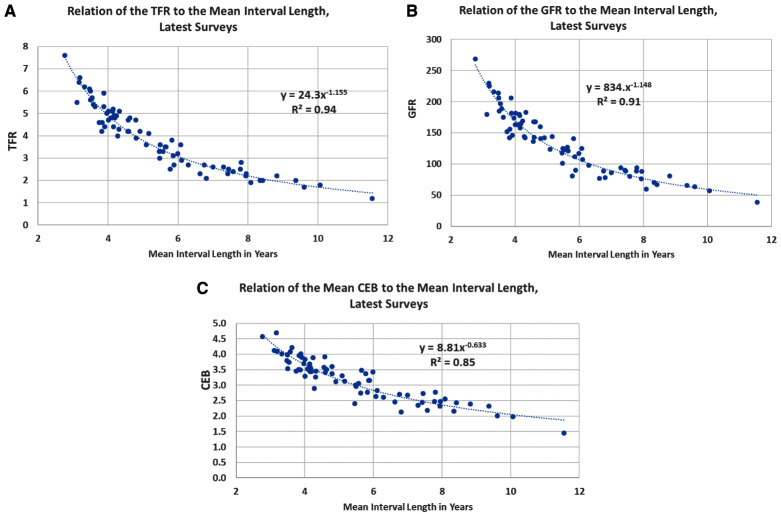
Relationships between three fertility measures and the open birth interval. **A**) Relation of the TFR to the mean interval length, latest surveys. **B**) Relation of the GFR to the mean interval length, latest surveys. **C**) Relation of the mean CEB to the mean interval length, latest surveys. TFR, total fertility rate; GFR, general fertility rate; CEB, children ever born.

The open birth interval and measures of fertility are closely connected. Across the 75 countries in the analysis, the R2 correlations with the mean open interval are 0.94 with the total fertility rate, 0.91 with the general fertility rate (GFR), and 0.85 with children ever born ( Figure 4). The relationship is curvilinear, with a diminishing slope at the longer intervals where the TFR approaches the replacement level. At the right tail of the distribution are countries that exhibit both low recent fertility and long open intervals. More individual women, mainly older ones, stopped childbearing after two children, up to 20 years ago.

The cross-sectional association can be augmented by examining the slope and degree of the association in each of the 59 countries with at least two surveys. Comparing the change in the TFR to the change in the mean open interval, we found a negative slope in 53 of the 59 countries, the other six showing either no TFR change or a positive slope. The median slope was -1.04 and the mean -1.18, for approximately a one-point decline in the TFR for each year of increase in the mean interval.

The closeness of the relationship is based heavily upon the common response by both measures to recent births: for the TFR the births often come from the previous three years; for the open interval they come from many births in those years as well as some in prior periods. Any downward or upward change in fertility as measured by the TFR or GFR must on average affect entries into the first interval, with its sizeable effect upon the mean.

### A stable state

Actual distributions for the open interval respond to numerous influences, but we can consider a growing population with a stable age distribution (involving fixed age-specific birth and death rates), with a steady flow of births annually and a stable open interval distribution. Period effects are then absent, and the shape of the open interval curve depends upon the fertility rate. With a low rate, new entries (births) would be few in relation to the entire group of women, just as the population age structure is more vertical under a low fertility rate. With low fertility the curve would start lower and remain flatter. A counter example is approximated by the Niger curve in Figure 3. Due to persistent high fertility it starts high and descends steeply.

### Modeling the open birth interval curve

**Figure 5.**
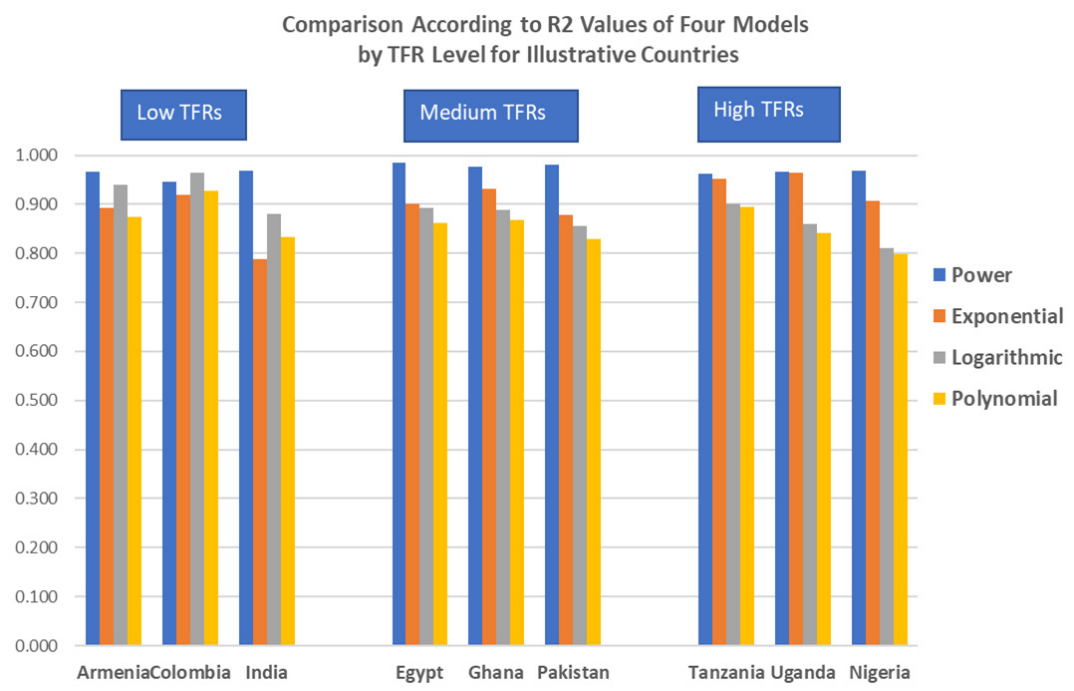
Comparison according to R2 values of four models by total fertility rate (TFR) level for illustrative countries.

The remarkable similarity of the shape of the open birth interval curve across countries invites efforts to find a few summarizing parameters. Limiting this to two parameters at most, we tested four options for best fit: power, exponential, logarithmic, and polynomial. Illustrative results appear in Figure 5 for three countries at three fertility levels; we found a power equation with two parameters to equal or exceed the fit of the other choices. Each bar in Figure 5 shows the R2 value between the curve and the fit. (Exact values are given in Table 3.

**Table 3.** Comparison of R2 values of four models by type and by total fertility rate (TFR) level.

TFR		Power	Exponential	Logarithmic	Polynomial	TFR
**Low**	Armenia	0.966	0.892	0.939	0.874	1.7
	Colombia	0.946	0.920	0.965	0.928	2.0
	India	0.968	0.789	0.880	0.834	2.2
**Medium**	Egypt	0.985	0.900	0.892	0.861	3.5
	Ghana	0.977	0.932	0.888	0.868	4.2
	Pakistan	0.980	0.878	0.856	0.829	3.8
**High**	Tanzania	0.962	0.953	0.901	0.894	5.2
	Uganda	0.966	0.965	0.859	0.841	5.4
	Nigeria	0.969	0.907	0.811	0.798	5.5

Calculations for the power equation start with the empirical distribution of the open birth interval in a country and follow the relation:

$$\text{y}={\text{ax}}^{\text{-b}}$$

That is, the percent of women in an interval equals “a” times the interval length in years taken to the “b” power.

**Figure 6.**
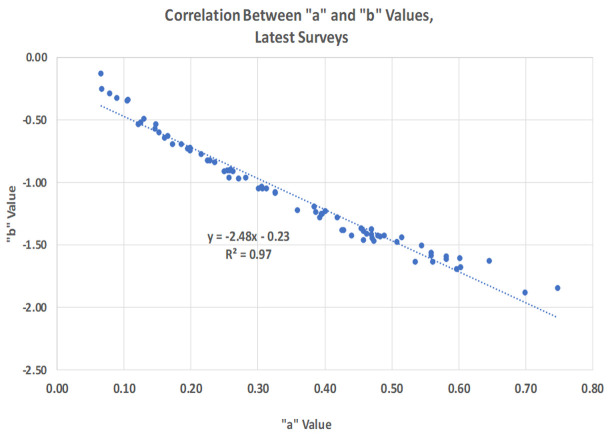
Correlation between “a” and “b” values, latest surveys.

The “a” parameter specifies the starting level of the curve, which is higher where more women fall into the first interval by being pregnant or in the first year after birth. It is highest in countries with high fertility, as in many sub-Saharan African countries. The “b” parameter captures the downward slope of the curve and is always negative; it too is largest where the curve starts high and descends rapidly across the first few intervals. It then levels off since women in all intervals add up to 100%. Therefore ( Figure 6), the two parameters tend to move together (R2 = -0.97). The “a” and “b” values both correlate at above 0.91 with the TFR and GFR and at above 0.88 with CEB (all positive for “a” values but all negatively for “b” values due to the downward slopes in the open interval distributions).

## Discussion

The open birth interval provides information not otherwise available on reproductive behavior. Past work to model the distribution of women according to the interval since the latest birth and its relation to fertility has been constrained for lack of empirical data. Information on the open interval distribution has now been generated across surveys covering most women in most developing countries. These show a systematic increase of age with interval length, and a systematic but much different pattern in change of parity. The data also show an intimate correlation between interval length and the three fertility measures of the TFR, GFR, and CEB. Moreover, the level and shape of the distribution of women by interval is captured with little error by an equation with two parameters.

Variation in the distribution is very large across countries, and in many it has changed considerably over time. The differences are most sensitive to the percentage of women in the first interval: women either pregnant or in the first year after birth. That percentage ranges from 10% to 47% of women, spanning low-fertility to high-fertility countries.

Determinants of the downward slope of the curve toward subsequent intervals include first the diminution of women in the general population with age, and then the various factors that extend birth-free time, including reduced fecundability and sexual frequency, mortality or other absence of the spouse, abortion, and contraceptive use.

The shape of the distribution is affected by both period and cohort effects, the former affecting all intervals at once, and the latter modifying the paths that successive groups of women follow across the intervals as they give birth in varying numbers and behavioral characteristics.

## Conclusion

Repeated surveys that, with a single question, trace changes in key patterns of childbearing, offer an efficient way to gain information for policies and programs directed to helping women defer or avoid unplanned births. They also apply to social policies related to women’s status; as the age of the youngest child increases, women’s freedom for roles and economic activities outside of the home is enlarged. The open birth interval can be a valuable resource for planning, and it deserves greater attention than it currently receives.

## Data availability

### Source data

The DHS Program prepares and makes available DHS survey data in the form of standard recode files in a range of file formats for use with several statistical software packages. All data used in this study are publicly available and free of charge upon registration at https://www.dhsprogram.com/Data/. A guide for how to apply for dataset access is available at: https://dhsprogram.com/data/Access-Instructions.cfm. The specific data files accessed were taken from each country listed in Table 4.

**Table 4.** Parameters "a" and "b" for power equations, TFR and GFR rates, and R2 value for fit with data.

	"a" Value	"b" Value	TFR	GFR	R2
Afghanistan 2015	0.440	(1.426)	5.3	175	0.956
Albania 2017-18	0.082	(0.283)	1.8	57	0.431
Angola 2015-16	0.603	(1.678)	6.2	216	0.970
Armenia 2015-16	0.121	(0.538)	1.7	64	0.968
Azerbaijan 2006	0.089	(0.328)	2.0	66	0.600
Bangladesh 2014	0.165	(0.630)	2.3	90	0.943
Benin 2017-18	0.673	(0.174)	5.7	197	0.912
Bolivia 2008	0.281	(0.963)	3.5	121	0.913
Brazil 1996	0.147	(0.538)	2.5	89	0.931
Burkina Faso 2010	0.581	(1.616)	6.0	206	0.961
Burundi 2016-17	0.748	(1.846)	5.5	180	0.923
Cambodia 2014	0.215	(0.779)	2.7	98	0.953
Cameroon 2011	0.426	(1.385)	5.1	180	0.977
Central African Republic 1994- 95	0.428	(1.388)	5.1	183	0.966
Chad 2014-15	0.597	(1.697)	6.4	230	0.954
Colombia 2015	0.129	(0.495)	2.0	70	0.947
Comoros 2012	0.392	(1.283)	4.3	142	0.981
Congo 2011-12	0.471	(1.453)	5.1	182	0.988
Congo Democratic Republic 2013-14	0.535	(1.641)	6.6	225	0.982
Cote d'Ivoire 2011-12	0.469	(1.423)	5.0	174	0.973
Dominican Republic 2013	0.172	(0.696)	2.5	89	0.977
Egypt 2014	0.271	(0.971)	3.5	127	0.985
Ethiopia 2016	0.544	(1.506)	4.6	156	0.925
Gabon 2012	0.359	(1.225)	4.1	144	0.947
Gambia 2013	0.561	(1.635)	5.6	185	0.974
Ghana 2014	0.386	(1.242)	4.2	143	0.977
Guatemala 2014-15	0.254	(0.904)	3.1	112	0.981
Guinea 2018	0.543	(0.152)	4.8	165	0.930
Guyana 2009	0.152	(0.602)	2.8	94	0.914
Haiti 2016-17	0.404	(0.124)	3.0	101	0.861
Honduras 2011-12	0.235	(0.844)	2.9	107	0.974
India 2015-16	0.125	(0.520)	2.2	81	0.968
Indonesia 2017	0.189	(0.737)	2.4	80	0.794
Jordan 2017-18	0.309	(1.065)	2.7	90	0.892
Kazakhstan 1999	0.105	(0.351)	2.0	67	0.819
Kenya 2014	0.400	(1.236)	3.9	141	0.929
Kyrgyz Republic 2012	0.257	(0.968)	3.6	125	0.985
Lesotho 2014	0.312	(1.050)	3.3	118	0.929
Liberia 2013	0.418	(1.282)	4.7	168	0.935
Madagascar 2008-09	0.396	(1.255)	4.8	168	0.958
Malawi 2015-16	0.701	(1.691)	4.4	158	0.914
Maldives 2016- 17	0.245	(0.904)	2.1	78	0.811
Mali 2012-13	0.559	(1.590)	6.1	214	0.967
Moldova 2005	0.078	(0.294)	1.7	55	0.747
Morocco 2003- 04	0.262	(0.912)	2.5	81	0.941
Mozambique 2011	0.458	(1.464)	5.9	206	0.975
Myanmar 2015- 16	0.198	(0.723)	2.3	77	0.932
Namibia 2013	0.305	(1.039)	3.6	125	0.948
Nepal 2016	0.146	(0.573)	2.3	88	0.970
Nicaragua 2001	0.258	(0.910)	3.2	117	0.906
Niger 2012	0.699	(1.881)	7.6	269	0.967
Nigeria 2018	0.559	(1.579)	5.3	182	0.940
Pakistan 2017-18	0.372	(1.217)	3.6	124	0.942
Papua New Guinea 2017	0.450	(1.350)	4.2	142	0.895
Paraguay 1990	0.395	(1.255)	4.7	160	0.943
Peru 2012	0.198	(0.746)	2.6	86	0.911
Philippines 2017	0.242	(0.888)	2.7	89	0.877
Rwanda 2014-15	0.646	(1.629)	4.2	142	0.832
Sao Tome and Principe 2008-09	0.457	(1.392)	4.9	164	0.952
Senegal 2017	0.764	(1.792)	4.6	152	0.917
Sierra Leone 2013	0.489	(1.428)	4.9	169	0.938
South Africa 2016	0.210	(0.809)	2.6	94	0.765
Swaziland 2006-07	0.307	(1.055)	3.8	137	0.946
Tajikistan 2017	0.281	(1.018)	3.8	141	0.939
Tanzania 2015-16	0.482	(1.437)	5.2	178	0.962
Timor-Leste 2016	0.384	(1.197)	4.2	136	0.913
Togo 2013-14	0.479	(1.425)	4.8	163	0.965
Turkey 2013	0.158	(0.637)	2.3	78	0.824
Uganda 2016	0.559	(1.568)	5.4	189	0.966
Ukraine 2007	0.066	(0.254)	1.2	39	0.798
Uzbekistan 1996	0.259	(0.900)	3.3	123	0.959
Viet Nam 2002	0.106	(0.341)	1.9	60	0.753
Yemen 2013	0.454	(1.373)	4.4	146	0.959
Zambia 2018	0.664	(1.675)	4.7	163	0.906
Zimbabwe 2015	0.470	(1.379)	4.0	144	0.931

### Code availability

Source code available from: https://github.com/kristinbietsch/OpenBirthInterval

Archived source code at time of publication: https://doi.org/10.5281/zenodo.4015278 ( Bietsch, 2020)

License: MIT

## Footnotes

^1 ^For the data used in this report we express appreciation to the ICF organization and its Demographic and Health Surveys Program.

^2 ^A useful overview is in Wikipedia, “One-Child Policy,” accessed May 18, 2020. https://en.wikipedia.org/wiki/One-child_policy

^3 ^The 2019 UN estimates for the group of Less Developed Countries shows 6.3% of women dying between exact ages of 15 and 50. At 11.1% the percentage is nearly doubled for the group of Less Developed Countries, most of them in sub-Saharan Africa. The rates accelerate by age. The percent of women dying within each age group, totaling 6.31% of the starting group at age 15, is shown in brackets for the following age groups: 15–19 (0.50), 20–24 (0.64), 25–29 (0.69), 30–34 (0.78), 35–39 (0.97), 40–44 (1.19), 45–49 (1.55).
