# Supplementary material for: The nature of the open birth interval distribution
Source: Gates Open Res. 2020 Oct 7;4:153. [Version 1] doi: 10.12688/gatesopenres.13177.1 (PMC8097239; doi:10.12688/gatesopenres.13177.1)
Supplement: Peer review history

# Peer review: Reviewer response for version 1

**DOI:** 10.21956/gatesopenres.14377.r29775

**Published:** 2021-05-04

**Authors:** Elizabeth Frankenberg

**Affiliations:** 1 Sociology Department, Carolina Population Center, University of North Carolina at Chapel Hill, Chapel Hill, NC, USA

It is relatively straightforward to ask a woman about the time that has elapsed since her latest birth and she is likely to answer that question relatively accurately. This paper addresses how answers to that question, aggregated from population-representative samples of women of reproductive age, differ across time and context, the extent to which summary statistics constructed from those answers vary by age and parity, whether the data can inform knowledge about current and past fertility in the populations in question, and how measures constructed from the data change over time for the same country. The premise, how data from one simple question can be used to characterize complex changes in population-level fertility over time, is intriguing. The authors have assembled an enormous amount of high quality publicly available data and the analyses are very thorough. The analyses will be of interest because fertility matters for the well-being of women, children, and families, and because fertility levels and patterns have changed so drastically over the past seventy-five years. Both technical demographers and those interested in population health should find the paper relevant to their work, but I believe it would be stronger with different framing and with a more parsimonious presentation of results. Some specific points are as follows:

- The review of past work, much of which was done in the 1960s and 1970s, could be condensed or moved to an appendix.
- Several times early on the relationships between open and closed intervals are mentioned. This may be of interest to technical demographers but it doesn’t seem the most effective starting point for making the case for the importance of the work overall.
- Figure 4, particularly in combination with Figure 1, does make the case for the importance of this work and might come earlier. To me, the regularity of the shape in Figure 1 combined with the ability to characterize that shape with only two parameters and the strong relation to measures of fertility levels is of the first order, but its impact is diluted by lots of details that come in between.
- For the case studies, which will be interesting for people who are not technical demographers, it might be more effective to use only two countries that differ dramatically in TFR at the latest survey and to provide some summary information about economic development level and the family planning programs in each country, perhaps Niger and India, assuming the data aren’t available for China.

Is the work clearly and accurately presented and does it cite the current literature?

Partly

If applicable, is the statistical analysis and its interpretation appropriate?

Yes

Are all the source data underlying the results available to ensure full reproducibility?

Yes

Is the study design appropriate and is the work technically sound?

Yes

Are the conclusions drawn adequately supported by the results?

Yes

Are sufficient details of methods and analysis provided to allow replication by others?

Yes

Reviewer Expertise:

demography, population health, fertility, surveys

I confirm that I have read this submission and believe that I have an appropriate level of expertise to confirm that it is of an acceptable scientific standard.

# Peer review: Reviewer response for version 1

**DOI:** 10.21956/gatesopenres.14377.r29773

**Published:** 2021-01-18

**Authors:** James F. Phillips

**Affiliations:** 1 Heilbrunn Department of Population and Family Health, Mailman School of Public Health, Columbia University, New York City, NY, USA

This paper, The nature of the open birth interval distribution, represents an important utilization of a vast array of contemporary survey data. Using 249 national surveys from 75 countries, the analysis represents an important step forward in fertility research. Research on birth interval dynamics spans a century of investigation, originating with analyses of historic records and extending to the papers amply cited in this review. Limitations of previous work are widely recognized as related to reliance on aggregated data that prevent rigorous statistical analyses of determinants or the complexity of birth history analyses that require hazard modeling. With this work, and research that it will inspire, the simplicity of open birth interval research can be marshaled for further work on fertility determinants that concern policy relevant variables, multi-level modeling, or hazard modeling of open intervals.  his paper is thus important, both for the scope of the data that are utilized and the potential for further research that this analysis invites.

Is the work clearly and accurately presented and does it cite the current literature?

Yes

If applicable, is the statistical analysis and its interpretation appropriate?

Yes

Are all the source data underlying the results available to ensure full reproducibility?

Yes

Is the study design appropriate and is the work technically sound?

Yes

Are the conclusions drawn adequately supported by the results?

Yes

Are sufficient details of methods and analysis provided to allow replication by others?

Yes

Reviewer Expertise:

Demographic research

I confirm that I have read this submission and believe that I have an appropriate level of expertise to confirm that it is of an acceptable scientific standard.

# Peer review: Reviewer response for version 1

**DOI:** 10.21956/gatesopenres.14377.r29774

**Published:** 2020-10-26

**Authors:** John Casterline

**Affiliations:** 1 Institute for Population Research, Ohio State University, Columbus, OH, USA

National demographic surveys often collect birth histories (partial or complete), and from this information, the length of the open birth interval is easily calculated. This manuscript analyzes data from 249 surveys in 75 countries conducted under the Demographic and Health Surveys program – a treasure trove of information! In fact, another 150+ surveys are available if one augments DHS with surveys conducted under other international survey programs, most notably the UNICEF MICS program. This body of data on the length of the open interval are overdue for systematic analysis such as provided here.   

  

1. What I find most interesting and most valuable in this manuscript, is the analysis in Figures 4 - 6 and Tables 3 and 4. This analysis: (i) assesses the ability of open birth interval distributions to predict standard measures of the level of fertility (TFR, GFR); (ii) examines how to represent the open interval distribution parsimoniously, e.g. no more than two parameters. This is an analysis of fundamental interest and value. 
2. In light of this – admittedly my own preferences – I am disappointed that the manuscript does not present the mean open birth interval (basic input for Figure 4). The distribution among the 249 surveys and 75 countries, summarized via some means (graphically and/or tabular), would be of considerable interest, in my view. Or, rather, it’s a striking absence.
3. I also wonder whether the authors have examined whether predicting the fertility measures (TFR, GFR) might be even more successful if the two parameters from exercise (ii) were used?

 

 Other points are as follows: 

  

1. I find little benefit from the two-page review of past approaches to open birth interval distributions provided in the Introduction. The capsule summaries of a diverse set of approaches, and heated methodological debates, I doubt will be comprehensible to most readers. (I speak for myself.)
2. Instead, what I think is key is to provide a good exposition of the pieces by Schmertmann in the late 1990s. This will require a bit of discussion, and possibly some formulae. In the Introduction, or possibly later, there should be some explanation of how the analysis in this manuscript connects to Schmertmann’s work.
3. Isn’t a crucial assumption when employing the open birth interval to compare across populations (e.g. within country over time, or between countries) that the historical trajectory of births in the years preceding the survey is the same? I don’t mean the level, rather the shape of the historical trend. This point is, finally, noted toward the end of the manuscript (pp. 11-12), and even then rather obscurely. Concern about the validity of this assumption is a main reason, I believe, for misgivings over the decades about analyzing open birth intervals alone (as against both open and closed birth intervals for, say, births occurring in the three or five years preceding the survey).
4. Data: DHS began in the late 1980s, not the late 1960s. Number of women: this exceeds 30K in a number of surveys, most notably the most recent survey in India (>600,000 women) but other surveys as well (e.g. the most recent three surveys in Colombia). There are many DHS surveys with less than 5000 respondents, but some of these may not be included in this analysis. Note that, contrary to the text, Table 4 does NOT provide a complete listing of surveys.
5. It is common to refer to “birth interval” and to say “by interval” when referring to 1 ^st^ birth interval, 2 ^nd^ birth interval, 3 ^rd^ birth interval, etc. Therefore it is confusing that this manuscript often uses “interval” to refer to various durations of the open interval (one year duration, two years’ duration, three years’ duration, etc.). For this reader, this was disorienting. For clarity, I urge the authors to adopt different terminology.
6. Figure 1: I do not find this helpful, except for overall visual impression. Individual surveys are indistinguishable.
7. Figure 2: I do not find these two sub-figures helpful. Beyond six or seven years, few women remain, as the text concedes. Yet these figures give essentially equal weighting to each duration. Moreover, I think percentaging in a different direction would be more to the point: by age-group, distribution of intervals by 8-10 duration categories maximum (1,2,3, . . .,7,8-9,10-14,15+ years); and, by parity, distribution of intervals by 8-10 duration categories.
8. Table 1: This is a detailed, space-consuming table that strikes me as tangential to the main purpose of this manuscript, which is to examine distributions of the open birth interval.
9. The paragraph on China on page 7 strikes me as a sidetrack.
10. Figure 3: Like Figure 1, the inability to distinguish clearly the various lines detracts from the value of this figure. Tabular presentation of the survey-by-survey distributions, by country, could be far more illuminating.
11. Pp. 9-10, discussion of direct determinants of open birth interval distribution. Post-partum behaviors – breastfeeding, abstinence – are not mentioned. (Although to be sure, sexual frequency is mentioned later in the manuscript, but not post-partum abstinence *per se*.) This is a major omission.
12. Figure 4: Please specify the reference period (I’m guessing three years preceding the survey).
13. Pg. 13 “Repeated surveys that, with a single question, trace changes in key patterns of childbearing, offer an efficient way to gain information for policies and programs directed to helping women defer or avoid unplanned births.” Doesn’t drawing inferences about unplanned births require classification of births as planned or unplanned? I don’t see how analysis of open birth interval distributions alone can do the trick.

Is the work clearly and accurately presented and does it cite the current literature?

Partly

If applicable, is the statistical analysis and its interpretation appropriate?

Yes

Are all the source data underlying the results available to ensure full reproducibility?

Yes

Is the study design appropriate and is the work technically sound?

Yes

Are the conclusions drawn adequately supported by the results?

Partly

Are sufficient details of methods and analysis provided to allow replication by others?

Yes

Reviewer Expertise:

Demography - fertility, reproductive health, family planning

I confirm that I have read this submission and believe that I have an appropriate level of expertise to confirm that it is of an acceptable scientific standard, however I have significant reservations, as outlined above.
